# A Disease Model for Wheezing Disorders in Preschool Children Based on Clinicians' Perceptions

**DOI:** 10.1371/journal.pone.0008533

**Published:** 2009-12-31

**Authors:** Ben D. Spycher, Michael Silverman, Juerg Barben, Ernst Eber, Stéphane Guinand, Mark L. Levy, Caroline Pao, Willem M. van Aalderen, Onno C. P. van Schayck, Claudia E. Kuehni

**Affiliations:** 1 Institute of Social and Preventive Medicine (ISPM), University of Bern, Bern, Switzerland; 2 Department of Infection, Immunity and Inflammation, University of Leicester, Leicester, United Kingdom; 3 Department of Pulmonology and Allergology, Children's Hospital St. Gallen, St. Gallen, Switzerland; 4 Respiratory and Allergic Disease Division, Paediatric Department, Medical University of Graz, Graz, Austria; 5 Pulmonology Unit, Department of Paediatrics, Geneva University Hospital, Geneva, Switzerland; 6 Allergy and Respiratory Research Group, Division of Community Health Sciences: GP Section, Edinburgh University, Edinburgh, United Kingdom; 7 Department of Paediatrics, Barts and the London Children's Hospital, London, United Kingdom; 8 Department of Paediatric Respiratory Medicine and Allergy, Emma Children's Hospital, Academic Medical Centre, Amsterdam, The Netherlands; 9 Department of General Practice, Maastricht University, Maastricht, The Netherlands; Ludwig-Maximilians-Universität München, Germany

## Abstract

**Background:**

Wheezing disorders in childhood vary widely in clinical presentation and disease course. During the last years, several ways to classify wheezing children into different disease phenotypes have been proposed and are increasingly used for clinical guidance, but validation of these hypothetical entities is difficult.

**Methodology/Principal Findings:**

The aim of this study was to develop a testable disease model which reflects the full spectrum of wheezing illness in preschool children. We performed a qualitative study among a panel of 7 experienced clinicians from 4 European countries working in primary, secondary and tertiary paediatric care. In a series of questionnaire surveys and structured discussions, we found a general consensus that preschool wheezing disorders consist of several phenotypes, with a great heterogeneity of specific disease concepts between clinicians. Initially, 24 disease entities were described among the 7 physicians. In structured discussions, these could be narrowed down to three entities which were linked to proposed mechanisms: a) allergic wheeze, b) non-allergic wheeze due to structural airway narrowing and c) non-allergic wheeze due to increased immune response to viral infections. This disease model will serve to create an artificial dataset that allows the validation of data-driven multidimensional methods, such as cluster analysis, which have been proposed for identification of wheezing phenotypes in children.

**Conclusions/Significance:**

While there appears to be wide agreement among clinicians that wheezing disorders consist of several diseases, there is less agreement regarding their number and nature. A great diversity of disease concepts exist but a unified phenotype classification reflecting underlying disease mechanisms is lacking. We propose a disease model which may help guide future research so that proposed mechanisms are measured at the right time and their role in disease heterogeneity can be studied.

## Introduction

Wheezing disorders in childhood are common and vary widely in clinical presentation and disease course (onset, remission and relapse). Within this syndrome, various phenotypes have been proposed, classified either by triggers of wheeze, into “episodic viral wheeze” triggered only by colds and “multiple-trigger wheeze” triggered also by other factors [1], [2], [3], or by time course into “early transient”, “persistent” and “late-onset” wheeze [4], [5]. Such phenotypes are being used in the study of risk factors [4], [6], [7], [8], [9], prognosis [10], [11], [12], [13] and response to treatment [2], [14], [15], and also increasingly in treatment recommendations and guidelines [2], [16], [17]. However it is unclear whether they represent distinct disease entities with separate aetiologies or rather different facets of the same disease. Because the underlying disease mechanisms are poorly understood it is difficult to define a biologically plausible classification of wheezing disorders.

Recently a “hypothesis-free” approach to define phenotypes has been explored [18], [19], [20], [21], [22]. This approach uses multivariate methods such as cluster analysis applied to a wide range of observed features including symptoms, signs or physiological measurements, in order to identify disease groups that might better reflect underlying biological pathways. These methods have yielded clusters (i.e. phenotypes) that are compatible with previous proposed phenotypes [19], [20]. However, clustering methods may distinguish groups regardless of whether they exist in the population as true entities or not. It is therefore essential to validate the output of these methods, i.e. to test whether the methods can distinguish between a homogenous and heterogeneous population, and whether they can identify the true disease entities existing within a heterogeneous population. Such a validation can, for instance, be done by artificially creating a heterogeneous population consisting of known disease entities and then applying the clustering method to data from this population to see whether the method can detect the heterogeneity and recover the true entities. This however requires a plausible model of disease consisting of predefined disease entities.

This study describes the creation of a plausible model of wheezing diseases in children, consisting of distinct hypothetical disease entities which would help in the validation of “hypothesis-free” methods. In order to obtain a disease model, we set up a small panel of clinicians familiar with paediatric wheezing disorders to agree on a set of disease entities for wheezing in preschool children based on their clinical experience.

We report here the qualitative part of this study which aimed to collect the initial views of the clinicians in the panel regarding the classification of wheezing disorders and, subsequently, to create a disease model by consent through structured discussions.

## Methods

### Selection of the Panel

The core group (authors BS, MS and CK) invited 9 clinicians from 4 countries Switzerland (CH), Austria (AT), the Netherlands (NL) and the UK of whom 7 participated (CH 2 participants, AT 1, NL 2, UK 2). These were selected from contacts known to members of the core group or brought to their attention through colleagues. All were clinicians working in primary care or paediatric pulmonology, who have seen many preschool children with wheeze, and whose individual views on asthma phenotypes were not previously known to the core group. The panel size was kept small enough to allow structured telephone discussions between members.

### Collecting Initial Views

The panel members were sent a questionnaire containing three questions. First, panel members were asked which view they most agreed with: (a) wheezing disorders in preschool children form a single, though highly variable, disease entity, (b) wheezing disorders in preschool children consist of different disease entities, or (c) undecided. In this context, a disease entity was defined as a wheezing disorder affecting a group of children which was distinct in aetiology from other wheezing disorders in other groups. The population of interest was defined as 0–5 year olds from the general population who are brought to a general practitioner with wheeze as a major symptom, and in whom wheeze caused by specific conditions such as cystic fibrosis, bronchopulmonary malformations or foreign body aspiration had been excluded. Second, respondents consenting to view (b) (above) were asked how many disease entities they thought existed, and third, for each of these entities, they were asked to provide a descriptive label and a bullet-point description of symptom patterns, physiological features, possible mechanisms, natural history and response to treatments.

The core group extracted the descriptive information on the proposed disease entities into tabular format. For this, feature categories (such as “shortness of breath” or “activities limited”) were formed based on the descriptions of all proposed entities. For each of the disease entities the table contained a feature profile with the entry ‘1’ if a particular feature was mentioned and ‘0’ if not. Using multiple correspondence analysis (MCA) [23], these feature profiles were graphically displayed in a plane representing the two main dimensions of variability. In this representation, points lying close together represent entities with similar feature profiles.

### Agreement on a Disease Model

The core group defined an initial model consisting of disease entities that had been proposed in a similar form by at least two respondents. The entities were described using a synthesis of the original bullet point descriptions by panel members. The panel was asked to propose and discuss changes to this model and agree on a final set of disease entities that could be justified by distinct pathophysiological mechanisms. These discussions were conducted by conference calls followed by e-mail correspondence. The phone conferences were recorded, abstracted and summarised to inform all panel members and for later reference.

## Results

### Initial Views and Propositions

**Figure 1:**
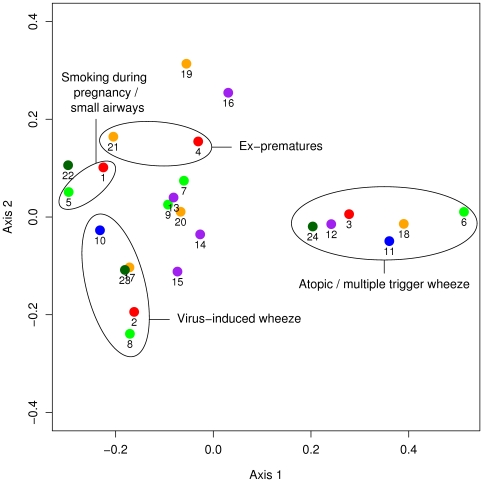
Graphical display of disease entities initially suggested by panel members. The figure shows the position of disease entities initially suggested by panel members along the two main axes of variability identified by multiple correspondence analysis. The position of each entity is determined by its feature profile (features used by the panel member to describe the entity) such that entities with similar features lie close together. The numbering follows that used in table 1 and entities suggested by the same panel member are coloured with the same colour. The ellipses indicate groups of entities which have similar label names but were suggested independently by different panel members. The fact that entities within the same ellipse lie close to each other indicates that when different panel members suggested similar labels they also gave a similar description of the corresponding entities. The initial disease model consisted of 4 entities representing the 4 ellipses.

Among the 7 respondents to the initial invitation, 6 held the view that these disorders consist of different disease entities, while one was undecided. Each of the 6 independently proposed and described 2–5 disease entities for classifying childhood wheezing disorders, amounting to an initial total of 24 disease entities (Table 1, Figure 1). MCA of the feature profiles showed that when different panel members suggested similar labels they also gave a similar description of the corresponding entities (Figure 1). For instance, among the 6 panel members subscribing to the multiple-disease view, all proposed an entity corresponding to wheeze associated with allergy or multiple triggers. These entities lie clustered together on the positive side of the main axis, axis 1, identified by MCA (Figure 1, ellipse on the right). Similarly, these members each suggested an entity corresponding to virus-induced (exclusive viral) wheeze with no other triggers (Figure 1, ellipse on the bottom left). Furthermore, two members suggested entities involving structural narrowing of airways because of maternal smoking during pregnancy, and two members suggested an entity comprising children born preterm. As a basis for further discussion, the core team therefore suggested an initial disease model consisting of these four entities using the labels: (i) allergic/multiple-trigger wheeze, (ii) exclusive viral wheeze, (iii) airway narrowing as a consequence of a developmental process, and (iv) ex-preterms.

**Table 1:** Classification of wheezing disorders in children aged 0-5 years as initially suggested by panel members.

Panel member	Disease entity nr	Descriptive label
1	1	Wheeze caused by tobacco smoke exposure (pre- or postnatal)
	2	Wheeze caused by viral and bacterial infections
	3	Allergic asthma
	4	Wheeze in ex-prematures
2	5	Airway narrowing as a consequence of an intrauterine process (smoking during pregnancy)
	6	Allergic (eosinophilic) asthma
	7	Acute viral bronchiolitis (in the first year of life)
	8	Viral induced bronchitis
	9	Happy wheezer
3	10	Episodic viral wheeze
	11	Multiple-trigger wheeze
4	12	Allergic wheeze (IgE mediated)
	13	Non-allergic asthma
	14	Infective wheeze
	15	Irritant exposure wheeze
	16	Exercise-induced wheeze
5	17	Virus-induced wheeze
	18	Atopic asthma
	19	Persistent wheeze (daily symptoms of wheeze; structural causes/malacia)
	20	Wheeze secondary to underlying condition
	21	Wheeze in ex-prematures, small airways
6	22	Wheeze associated with bronchial secretions
	23	Viral wheeze, episodic to persistent
	24	Viral wheeze associated with atopic disposition
7	-	Undecided

### Discussions and Agreement on a Disease Model

There was general agreement among members that an allergic form of wheeze associated with interval symptoms should be distinguished from wheeze occurring only during viral infections, i.e. (i) and (ii). The panel members also agreed that the entities (iii) and (iv) might be subsumed to a single entity, because structural airway narrowing was considered to be the major cause for viral wheeze in this age group. Both smoking during pregnancy and pre-maturity could be regarded as predisposing factors for airway narrowing and should not define separate disease entities.

There was a consensus that atopy and structural airway narrowing represented two mechanisms of current wheezing. Proposing further mechanisms and phenotypic features that would distinguish these from other mechanisms was perceived as highly speculative for the following reasons.

1. Although triggers are commonly used to distinguish phenotypes, they may not be specific for individual mechanisms. For instance, wheeze occurring only during viral infections may be due to structurally narrow airways. However, almost all wheezy children in this age group have symptoms during viral infections, suggesting that other mechanisms are involved.
2. There is a lack of feasible measurements in this age group that would distinguish children with different outcomes in later childhood. For instance, in the first few years of life, children who later develop allergic wheeze are clinically very similar to children who only wheeze during viral infections. One can only speculate whether the mechanisms for viral wheeze in these two groups differ.
3. It is unclear to what extent this age group is also affected by non-allergic, multiple-trigger wheeze that might be indicative of different mechanisms.

However, for the purpose of this study, the panel members agreed on a final set of 3 entities (Table 2) that plausibly explain the observed heterogeneity of childhood wheezing disorders: Allergic wheeze, non-allergic wheeze due to structural airway narrowing and non-allergic wheeze due to increased immune response to viral infections. These entities represented three mechanisms that could contribute to recurrent wheezing diseases in young children.

**Table 2:** Disease model for wheezing disorders in children aged 0–5 years as agreed by panel.

Descriptive label		A) Allergic wheeze	B) Non-allergic wheeze due to structural airway narrowing	C) Non-allergic wheeze due to increased immune response to viral infections
**Symptoms and triggers**	**Symptoms**	Wheeze, cough, breathlessness, nocturnal cough	Wheeze, cough, breathlessness	Wheeze, cough, breathlessness
	**Symptom pattern**	Episodic, often with interval symptoms; severity and duration of episodes highly variable; perennial	Episodic; episodes short (<2 wks) and variable in severity; occurring mainly during the cold season	Episodic; episodes short (<2 wks) and variable in severity; occurring mainly during the cold season
	**Potential triggers**	Viral infections, allergens, ETS, pollution	Mainly viral infections, ETS, possibly exercise	Viral infections only
**Physiological features**	**Lung function, reactivity**	Normal LF when well controlled, decreased otherwise; chronically reduced in persistent severe asthma; positive BDR; BHR	Reduced LF; BHR	Reduced LF during exacerbations, normal if symptom-free; BDR positive only during exacerbations
	**SPT**	Positive (aeroallergens)	Negative	Negative
	**Other**	Increased exhaled NO; eosinophils (BAL); airway remodelling (biopsy)	Normal exhaled NO; neutrophils (BAL)	Normal exhaled NO; neutrophils (BAL)
**Aetiology**	**Mechanisms**	Allergic inflammation in combination with other causes of increased responsiveness to viral infections	Congenital narrowing of airways causing increased responsiveness to viral infections	Increased immune responsiveness to viral infections not related to atopy; possibly a maturational defect
	**Risk factors**	Parental atopy or asthma (strong genetic component), high exposure to allergens and high frequency of viral infections in early life	Smoking during pregnancy, premature birth, genetic predisposition	Genetic predisposition, premature birth, increased frequency of viral infections
**Natural history**	**Onset**	Atopic predisposition at birth; early onset; non-viral triggers usually >4 yrs	In infancy	0–3 years
	**Persistence and prognosis**	Tends to persist into adolescence and adulthood; can lead to irreversible airway obstruction	Tendency to remit by 5 years; relapse and/or reduced lung function in later life possible	Tendency to remit by 5 years; relapse and/or reduced lung function in later life possible
**Response to treatment**		Good response to ICS though not disease modifying; good response to bronchodilators	No response to ICS; poor response to bronchodilators	Poor response to ICS; bronchodilators can help

Abbreviations: BAL bronchoalveolar lavage; BDR bronchodilator response; BHR bronchial hyperresponsiveness; ETS environmental tobacco smoke; ICS inhaled corticosteroids; LF lung function; NO nitric oxide; SPT skin prick test.

Although there was some disagreement concerning acute viral bronchiolitis, panel members agreed that none of the other entities they had originally proposed should be considered as separate disease entities. The consensus was, that some of them represented wheezing secondary to underlying conditions with clear diagnoses (7, 19, 20 in Table 1), while others were limited to particular exposures (15), were poorly defined and outdated (9) or were a general sign of inflammation (22).

## Discussion

In this study we aimed to agree on a disease model for wheezing disorders in young children consisting of distinct disease entities each based on plausible mechanisms. We wanted this model to be developed and agreed upon by clinicians who encounter the full range of clinical presentations of these disorders, working in primary, secondary, and tertiary care. The model is the first step in a validation study of statistical methods used to identify subgroups of disease from epidemiological data.

### Opinions of Panel Members

First, we found that the majority of clinicians (6/7 in our sample) supported the view that wheezing disorders in preschool children consist of several different disease entities. Second, we found a broad range of concepts and definitions for different forms of childhood wheeze. Among the disease entities proposed by the 6 panel members subscribing to a multiple-disease view, only a few entities were similar across two or more physicians, the best agreement being found for the two entities described as “allergic wheeze associated with interval symptoms” and “exclusive viral wheeze occurring only during viral infections”. There was no agreement among panel members on the potential number of disease entities. Third, clinicians' concepts of disease mechanisms underlying wheezing disorders were vague. Except for allergic wheeze, which represents the classical asthma phenotype, all panel members were reluctant to define entities in terms of explicit mechanisms, and perceived this task as highly speculative. Fourth, it became clear that the clinical information that might be used to distinguish different mechanisms is limited in early childhood. For instance, non-viral triggers that may be important in later years are less relevant (aeroallergens) or cannot be assessed (exercise) in early childhood. Also, the possibilities for physiological measurements are restricted in the first few years of life.

### Comparison with Literature

To our knowledge, this is the first study describing current views of clinicians on phenotypes of wheeze in preschool children, and the first which has tried to obtain a consensus among them in order to propose a disease model. The final model proposed by the panel, and the main mechanisms suggested reflect current discussions in the literature.

#### Allergy

Allergic sensitisation is associated with persistent wheeze in childhood but not with early transient wheeze [24], [25]. There is evidence that allergic sensitisation can begin in infancy, in particular sensitisation to food allergens [26], [27], [28], however, incidence increases markedly after the age of 2–3 years mainly due to sensitisation to aeroallergens [26]. In children who develop atopic asthma onset of sensitisation tends to be early [29].

#### Structurally narrow airways

Evidence suggests that poor lung function in infancy is associated with subsequent early childhood wheezing [4], [30]. Flow limitation can be caused by decreased airway dimensions and/or altered airway wall mechanics [31]. Factors that can lead to such changes in airway structure and/or function include maternal smoking during pregnancy [32] or preterm birth, even in the absence of neonatal respiratory disease [33], [34].

#### Increased immune responsiveness to viral infections

Wheeze in infancy is predominantly triggered by viral infections [35]. The majority of children experience respiratory viral infections in the first years of life and responses vary widely from asymptomatic disease in some children, through mild upper respiratory illness in many to severe lower respiratory disease in a minority of infants [36]. Host specific factors associated with the severity of this response include mechanical and immunologic factors such as an imbalance between Th1 and Th2 type responses [37], [38].

The disease model proposed by the panel of clinicians is therefore not novel with respect to the hypothesised mechanisms. It is however original in the selection of these particular mechanisms as the main sources of heterogeneity in preschool wheezing disorders. Other expert-driven attempts to distinguish phenotypes of wheeze have mainly been based on long-term temporal patterns (early transient, persistent and late onset wheeze) [4], [5] or main triggers (viral wheeze, multiple-trigger wheeze) [1], [2], [3].

### Strengths and Limitations of Our Approach

Our approach brought together a wide range of initial views and concepts from clinicians who encounter the full clinical spectrum of wheezing disorders in young children. The panel was heterogenous, composed of physicians from four different countries, including both paediatric pulmonologists and general practitioners, yet small enough to allow a co-ordinated discussion and an agreement on a final disease model. We included people who have a broad clinical experience and research interests, but who had not been performing research on phenotypes of childhood wheeze nor were closely related to a group which had done this. Therefore, our results reflect a broad range of different opinions from specialists and generalists across different European countries.

Limitations of this study include small sample size and reliance on subjective views of individuals. Clearly, sample size is too small to represent the full spectrum of views amongst clinicians at large, and we cannot know the extent to which the list of initially proposed disease concepts is exhaustive. Thus, inclusion of more clinicians might have resulted in more initial disease entities, and perhaps in a different final disease model. However, our study did not aim to obtain a representative consensus view for the wider population of clinicians; rather it was an attempt to generate hypotheses for further research and discussion. Also, our conclusions are not based on objective data, but on the subjective views of clinicians. Clinician's perceptions represent a synthesis of information from a wide range of sources including first hand clinical observations, but also published literature. We wanted to combine this information to generate a model for wheezing disease. The study was designed as a qualitative and exploratory study and not as a confirmatory study that would require large samples of preferably objective data.

### Conclusions and Suggestions for Further Research

Our study suggests that there is wide agreement among clinicians that preschool wheezing disorders consist of several disease entities, but that there is less agreement as to how many and which these are. Although a great diversity of specific disease concepts exists there is much uncertainty regarding the biological mechanisms that could justify them. A major problem is that the clinical features observed in this young age group are not specific for later disease course and physiological measurements are difficult.

We propose a disease model for wheezing disorders in preschool children comprising three entities linked to specific mechanisms: a) allergic wheeze, b) non-allergic wheeze due to structural airway narrowing and c) non-allergic wheeze due to increased immune response to viral infections. Further research is needed to determine whether or not these mechanisms are major causes of wheezing in early childhood. The proposed model may be useful for designing future studies so that hypothesised mechanisms are measured at the right time. Few epidemiological studies to date have collected data prospectively and at frequent intervals on the early development of atopic sensitisation (for instance [39]) or on factors related to increased responsiveness to viral infections in early life [4], [30], [40], [41]. Prospective studies including repeated measurements of lung mechanics and physiology during infancy and childhood in representative cohorts of children are almost entirely lacking. There is an urgent need to develop non-invasive measurements of lung function that can be applied at all ages. Only this will allow these hypothesised entities, assuming that they do exist to be disentangled.

If confirmed, these different causes of wheezing may have important implications for treatment. Wheeze due to structural airway narrowing might be a self limited disease, the natural course of which may not be influenced by treatment. These children are likely to outgrow their symptoms as their airways increase in size with growth. However, because relative differences may persist throughout life, relapse may occur in later adulthood as elastic recoil decreases and lung function declines. The greatest potential for disease modification by treatment is certainly for allergic wheeze, but also, potentially, for the hypothesised entity “non-allergic wheeze due to increased immune responses to viral infections”.

As our study has shown, there is a clear need for a standardisation of phenotype definitions. Ultimately such a classification should be based on an understanding of the pathogenesis of different disease entities.
